# Opioid Use Following Spine Surgery: Strategies for a Multimodal Approach To Pain Management

**DOI:** 10.1007/s11916-025-01428-6

**Published:** 2026-01-03

**Authors:** Leonie Erbeldinger, Benjamin Martens, Richard D. Urman, Markus M. Luedi

**Affiliations:** 1 Department of Anaesthesiology, Rescue- and Pain Medicine, Cantonal Hospital of St. Gallen, St. Gallen, Switzerland; 2 Department of Orthopedic Surgery, Cantonal Hospital of St. Gallen, St. Gallen, Switzerland; 3 Department of Anesthesiology, College of Medicine, The Ohio State University, Columbus, OH USA; 4 Department of Anaesthesiology and Pain Medicine, Inselspital, Bern University Hospital, University of Bern, Bern, Switzerland

**Keywords:** Opioids, Opioid misuse, Spine surgery, Back pain, Postoperative pain management

## Abstract

**Purpose of Review:**

Chronic back pain is highly prevalent and closely associated with opioid misuse, particularly in patients undergoing spine surgery. Optimizing opioid prescribing practices and advancing alternative treatment modalities is critical to reduce opioid-related morbidity and mortality.

**Recent Findings:**

Despite their well-documented risks—including misuse, adverse effects, and detrimental impacts on postsurgical outcomes—opioids remain the most commonly prescribed analgesics for back pain. Spine surgery, meanwhile, is frequently followed by intense postoperative pain due to central sensitization, which often necessitates opioid use and complicates pain management. This review provides an overview of current literature on opioid prescribing trends and alternative therapies for patients undergoing spine surgery.

**Summary:**

Following spine surgery, non-opioid pharmacologic agents and nutraceuticals can enhance analgesia and reduce opioid consumption. While erector spinae plane blocks and neuraxial techniques offer transient pain relief, their efficacy is limited by duration and potential risks. Spinal cord stimulation may benefit selected patients with back pain, although its opioid-sparing effects remain uncertain. Opioid prescribing should be limited to breakthrough pain and integrated into structured tapering strategies. Optimizing postoperative analgesia in spine surgery requires a multimodal approach, interdisciplinary collaboration, and individualized prescribing—potentially guided by emerging tools such as pharmacogenomic testing.

## Introduction

Chronic back pain is a globally prevalent condition, with an estimated 570 million cases worldwide in 2019It represents a leading cause of disability and a major driver of emergency department visits due to associated symptoms [1, 2]. Back pain is also a primary indication for spine surgery, making it one of the most frequently performed procedures and among the most expensive surgical interventions globally [3]. Consequently, its burden on global healthcare systems and quality of life is substantial.

Chronic back pain is strongly linked to long-term opioid use, placing patients at elevated risk for adverse effects and opioid dependence [4–7]. In the context of the current opioid epidemic, substantial research has focused on the role of opioid therapy in this population. Understanding the underlying mechanisms of opioid dependence, evaluating its impact on clinical outcomes, and identifying effective non-opioid pain management strategies are essential to mitigate opioid-related harm. Notably, the interplay between spine surgery and opioid use represents a central issue from both clinical and economic perspectives. 

This narrative review provides a comprehensive synthesis of the current literature on opioid use in the setting of chronic back pain and spine surgery. We aim to elucidate the bidirectional relationship between surgical intervention and opioid consumption, and to discuss evidence-based strategies to reduce opioid prescribing. Addressing these challenges is vital, and we emphasize the need for enhanced awareness and education among healthcare providers. 

## Opioid-related Prescription Challenges

### Definitions and Epidemiology of Opioid Misuse

Opioids are synthetic compounds derived from opium, the dried latex of the poppy plant (Papaver somniferum). Although natural opium has been used for over 5000 years, systematic pharmacologic exploration of its derivatives began in the 19th century. These substances are primarily employed for their potent analgesic and sedative properties. However, they are also misused recreationally to induce euphoria, making them a significant component of the global illicit drug market [8].

According to the International Classification of Diseases (ICD-11), opioid dependence is defined as a substance use disorder marked by a compulsive urge to consume opioids despite adverse consequences, often accompanied by tolerance and withdrawal symptoms [9]. Over recent decades, the global prevalence of opioid use disorder has increased steadily [10]. As of 2023, approximately 5.7 million individuals in the United States were diagnosed with opioid use disorder, with over two-thirds of drug overdose deaths attributed to synthetic opioids [11]. A deeper understanding of opioid pharmacology is essential to inform strategies aimed at mitigating this public health crisis. 

### Mechanism of Action of Opioids

Opioids exert their effects by binding to specific G-protein-coupled receptors—mu (µ), kappa (κ), and delta (δ)—located in the central and peripheral nervous systems. The analgesic effect primarily results from decreased neuronal excitability through calcium channel inhibition and increased potassium conductance, ultimately reducing the release of pro-nociceptive neurotransmitters [12, 13].

Each receptor subtype contributes to analgesia via distinct mechanisms: kappa receptors mediate peripheral analgesia, delta receptors modulate spinal pain pathways, and mu receptors are responsible for the most potent analgesic effects. All receptor types are involved in supraspinal modulation of pain [12]. An additional receptor, opioid receptor-like 1 (ORL1), is currently under investigation for its role in hyperalgesia and neuropathic pain, though its clinical significance remains unclear [13]. 

The widespread distribution of opioid receptors explains their broad pharmacologic effects beyond analgesia, including respiratory depression, pruritus, constipation, euphoria, and anxiolysis. These latter effects contribute significantly to the development of opioid misuse and addiction [13]. 

### Opioid Misuse

Multiple biological mechanisms contribute to opioid tolerance and misuse. At the cellular level, chronic opioid exposure leads to neuroadaptive changes, such as normalization or overactivation of downstream signaling pathways—particularly involving cyclic adenosine monophosphate (cAMP). Additionally, long-term use alters receptor expression and availability, as well as calcium channel regulation, culminating in both tolerance and opioid-induced hyperalgesia. Increased production of endogenous pain facilitators, such as cholecystokinin, further diminishes opioid efficacy over time [12, 14].

Opioids also act on the mesolimbic dopamine system, elevating dopamine levels in the nucleus accumbens to produce euphoria and reduce anxiety, reinforcing drug-seeking behavior. Chronic use impairs prefrontal cortex function, weakening impulse control and fostering compulsive use. Psychological factors—including psychiatric comorbidities and risk-prone personality traits—further predispose individuals to opioid addiction, often through self-medication pathways [12, 15].

The intersection of tolerance, dependence, and addiction creates a high-risk scenario for overdose and fatal outcomes, underscoring the urgency of optimizing prescription practices [12].

### General Recommendations for Opioid Use

Safe opioid prescribing begins with thorough risk assessment. Clinicians should review the patient’s medical and prescription history, ideally using a Prescription Drug Monitoring Program (PDMP), and coordinate care with other providers, particularly pain specialists. Before initiating opioids, clinicians must provide patient education regarding treatment goals, expected duration, and risks.

Opioids should be prescribed at the lowest effective dose, for the shortest feasible duration, and restricted to breakthrough pain rather than baseline analgesia. Every attempt should be made to use non-opioid medications to supplement opioids when the latter are deemed necessary [16, 17].

Throughout the duration of opioid therapy, the patient should undergo regular monitoring. During opioid therapy, ongoing evaluation is critical. Pain intensity can be monitored using validated tools such as the Numeric Rating Scale, while urine drug testing helps detect misuse or non-compliance [16]. 

Once acute pain resolves, opioids should be gradually tapered or discontinued. Tapering strategies—such as dose reduction, adjunctive medication use, or opioid rotation—should be tailored to individual needs. Although tapering can reduce tolerance, dependence, and adverse effects, patients remain at risk for relapse or overdose, particularly if they lose follow-up or turn to illicit substances. In cases where tapering fails, long-acting opioid agonist therapy (e.g., methadone, buprenorphine) may improve function and reduce illicit use, though it may not eliminate dependence [17]. 

Overall, opioid therapy must be personalized and closely monitored, addressing both biological and psychosocial factors. 

### Pharmacogenomics

Pharmacogenomic research aims to individualize opioid therapy by identifying genetic polymorphisms that influence drug metabolism, efficacy, and risk of adverse events [18]. Key genes include those encoding the mu-opioid receptor, cytochrome P450 2D6, and catechol-O-methyltransferase [16, 19, 20]. Global pharmacogenomic databases are emerging to inform clinical decision-making.

Despite promising results, routine implementation of pharmacogenomic testing remains limited due to high costs and a lack of standardized clinical guidelines [21]. Additionally, factors such as sex, socioeconomic status, and comorbidities also influence opioid response and must be considered [16, 20, 22]. 

### Barriers for Use

Ear of addiction is the most frequently cited barrier to postoperative opioid use, affecting up to 75% of patients. Other concerns include fear of adverse effects, disease progression, social stigma, and distrust in non-opioid alternatives [23]. These misconceptions are widespread among patients and clinicians alike and are often exacerbated by media coverage that underrepresents the legitimate role of opioids in acute pain management.

Effective patient education and shared decision-making are essential to counteract misinformation and improve adherence to pain management plans [24]. 

## Opioid Use in the Context of Spine Surgery

### Burden of Back Pain and Opioid Prescribing Trends

As discussed in the introduction, chronic back pain represents a substantial economic and healthcare burden worldwide. It contributes to increased healthcare utilization, absenteeism from work, and has remained the leading cause of years lived with disability for decades across both high- and low-income countries [25, 26]. 

Although nonsteroidal anti-inflammatory drugs (NSAIDs) are recommended as first-line therapy, opioids continue to be the most frequently prescribed medications for chronic back pain [27–29]. While opioid prescribing in emergency departments has declined in recent years, more than half of patients presenting with back pain still receive at least one opioid. Notably, patients who are older than 44 years, have private insurance, or higher socioeconomic status are more likely to be discharged with opioid prescriptions [30]. These findings suggest that prescribing behaviors may be influenced by non-clinical factors, potentially restricting access to guideline-concordant care. 

Patients are often willing to accept the risks associated with opioids in exchange for adequate pain relief. However, physical dependence remains one of the most feared complications of opioid therapy in chronic back pain management [31]. This widespread use of opioids creates significant challenges in the perioperative care of patients undergoing spine surgery. 

### Preoperative Opioid Use

Before undergoing spine surgery, approximately 8.7% of patients meet criteria for persistent opioid use, while only 45% are opioid-naïve. These rates are relatively independent of symptom duration [4, 32].

Key predictors of persistent preoperative opioid use include a high burden of comorbidities, certain demographic factors, the presence of radicular symptoms (e.g., leg pain), and prior spine surgery [4, 33, 34]. In particular, the association with previous surgeries suggests that not all operations result in adequate pain control and may contribute to ongoing opioid use. 

Differences in symptomatology also affect opioid utilization [29]. Patients with radiculopathy tend to use opioids more frequently than those with neurogenic claudication, likely due to the temporary relief obtained from rest in the latter condition. Additionally, individuals with degenerative disc disease are more likely to receive opioids compared to those with spinal deformities, canal stenosis, or spondylolisthesis [32].

### Perioperative Opioid Use

Perioperative opioid administration is associated with an increased risk of prolonged postoperative opioid dependence and a higher incidence of complications such as infections, gastrointestinal dysfunction, and reoperations—leading to longer hospital stays. Paradoxically, perioperative opioid use may shorten the time to return to work [35].

When analyzing specific agents, higher intraoperative doses of remifentanil do not appear to increase postoperative opioid consumption or reported pain intensity despite the concern of postoperative hyperalgesia, supporting its use as a safe intraoperative analgesic option [36, 37]. 

### Postoperative Opioid Use

Among patients with a history of opioid use prior to surgery, approximately 50% continue opioid consumption for at least two years postoperatively. The most significant predictor of persistent use is concurrent administration of high-dose benzodiazepines. Alarmingly, up to 10% of opioid-naïve patients develop new-onset persistent opioid use after spine surgery [4, 32].

One proposed mechanism for this phenomenon is central sensitization, wherein surgical manipulation of the spinal cord leads to persistent mechanical hypersensitivity and chronic pain [38]. This may explain why patients undergoing multiple surgeries at the same spinal level are at elevated risk for persistent pain and opioid use [4].

Surgical technique and location also influence opioid requirements. Spinal fusion procedures are associated with lower postoperative opioid consumption compared to non-fusion surgeries, and lumbar surgeries confer a higher risk than cervical procedures [35]. 

These observations underscore the importance of stringent surgical indications based on evidence-based criteria and thorough risk-benefit assessments in candidate selection [39]. 

### Surgical Outcome

Postoperative pain following spine surgery frequently encompasses both nociceptive and neuropathic components. Tissue trauma contributes to nociceptive pain, whereas nerve injury causes neuropathic pain, often resulting in a complex, mixed pain phenotype [40]. Adequate pain control is vital to avoid delayed recovery, psychological distress, and increased healthcare costs.

A key concern is chronic postsurgical pain (CPSP), which affects up to 50% of spine surgery patients. CPSP is largely driven by central sensitization, resulting in long-lasting changes in neural pathways and heightened pain perception [40]. 

Favorable predictors of improved postoperative pain outcomes include younger age, lower preoperative pain intensity, fewer comorbid conditions, and greater psychological resilience. Interestingly, symptom duration prior to surgery appears to have minimal influence on postoperative pain [41]. 

In general, patient-reported outcome measures (PROMs) improve significantly following spine surgery [4]. Although individuals with preoperative opioid dependence have lower baseline PROM scores and face higher risks of complications and prolonged hospitalization, they still derive meaningful improvements postoperatively [6, 35, 42, 43]. With the exception of psychological domains—such as depression or sleep disturbances—this subgroup often achieves outcomes comparable to opioid-naïve patients. 

Surprisingly, there is no observed difference in outcomes between opioid users managed through pain clinics and those who are not, suggesting the need for further investigation into individualized support strategies [6]. 

Despite these gains, postoperative opioid use often persists, reflecting the complex interplay between surgery, pain, and pharmacologic management—and reinforcing the need for alternative analgesic strategies [4, 32]. 

## Alternative Approaches for Pain Management in Spine Surgery

### Drug and Supplement Regimes

In the context of postoperative pain management, combining agents with different mechanisms of action improves analgesia and reduces adverse effects. This approach is endorsed by the World Health Organization [43]. After spine surgery, first-line pharmacologic therapy consists of scheduled (rather than as-needed) non-opioid analgesics [44]. The most effective regimen includes acetaminophen and an NSAID, supplemented by either an adjuvant such as duloxetine or an alpha-2 agonist, or by gabapentin [45–47]. Among these combinations, regimens including duloxetine or similar agents have demonstrated the most significant reductions in opioid consumption [48]. 

Another promising non-opioid pain reliever in Phase 3 clinical trials is suzetrigine. It selectively targets the NaV1.8 sodium channel, found only in peripheral sensory nerves. By avoiding the brain and spinal cord, it interrupts pain signals without affecting the central nervous system, minimizing the risk of addiction. Clinical studies show suzetrigine significantly reduces acute pain compared to placebo and causes fewer side effects – particularly less postoperative nausea and vomiting - than opioid treatments, offering a safer, effective alternative [49, 50]. 

In contrast, intravenous lidocaine infusions have not shown consistent benefits in reducing either postoperative pain or opioid use and therefore are not recommended [48].

Growing attention is being directed toward non-pharmacological supplements with analgesic potential [51]. For example, magnesium has been shown to reduce intraoperative anesthetic requirements and postoperative opioid consumption. Vitamin B complex in combination with NSAIDs has demonstrated superior efficacy over NSAIDs alone for back pain relief. Curcumin, a bioactive compound from turmeric, reduces postoperative pain more effectively than placebo—and its efficacy is further enhanced when co-administered with boswellia, a resin-derived herbal extract. These supplements are not only effective but also affordable and generally well tolerated, making them promising adjuncts in multimodal pain strategies [43]. 

Enhanced Recovery After Surgery (ERAS) protocols represent a comprehensive, multidisciplinary approach to perioperative care, aimed at reducing postoperative complications, improving outcomes, and minimizing opioid use. In spine surgery, ERAS includes evidence-based pharmacologic management, early mobilization, physical therapy, patient education, and optimized wound care [52]. However, guideline development for ERAS protocols in spine surgery—particularly for cervical and thoracic procedures—remains limited [53]. Further high-quality studies are needed to expand and refine ERAS recommendations for this population [54]. 

### Regional Interventions

The erector spinae plane (ESP) block is an ultrasound-guided, interfascial nerve block that has gained popularity in spine surgery. This technique involves a single-shot injection of local anesthetic adjacent to the transverse processes at the surgical level. ESP blocks have been shown to reduce the need for intraoperative fentanyl from 90% to 13% and significantly decrease postoperative pain for up to 12 h [54]. Hcwith no significant difference in pain scores observed at 24 h. As a single-shot technique, its efficacy depends on the pharmacokinetics of the local anesthetic used. Research is ongoing to assess the utility of continuous ESP block catheters for extended analgesia. [48].

Although ESP blocks are generally safe, rare but serious complications—such as infection, pneumothorax, or vascular injury—can occur, highlighting the importance of experienced operators and vigilant monitoring [54].

A related strategy is direct wound infiltration with local anesthetics, which, like ESP blocks, reduces intraoperative opioid requirements but is limited by its short duration of action [48, 54]. 

### Spinal Cord Stimulation (SCS)

Spinal cord stimulation (SCS) involves the epidural placement of electrical leads to modulate nociceptive transmission. While its exact mechanism remains unclear, SCS is thought to inhibit central sensitization and disrupt pain signaling. When used intraoperatively, SCS may attenuate the central sensitization triggered by spinal cord exposure [38, 42].

Postoperatively, SCS has demonstrated high success rates for treating chronic radicular pain, especially following cervical or lumbar procedures. However, its efficacy is more limited in patients with direct spinal cord pathology or central nervous system involvement. Importantly, data remain inconclusive regarding the ability of SCS to meaningfully reduce long-term opioid use [38, 42].

### Neuraxial Interventions

Neuraxial interventions include both intrathecal and epidural routes, delivered either via single-shot injections or continuous infusion pumps. Commonly administered agents include local anesthetics, corticosteroids, clonidine, and opioids [42, 54].

Evidence for single-shot neuraxial techniques in spine surgery is inconsistent. While some studies suggest reductions in postoperative opioid consumption, their analgesic effects are short-lived, and improvements in pain scores are not uniformly observed [48, 54]. These limitations may be overcome by continuous delivery methods. 

For example, epidural infusions of bupivacaine combined with fentanyl significantly reduce postoperative opioid requirements. Likewise, intrathecal and epidural infusion pumps can diminish or even eliminate opioid dependence in selected patients [42, 55]. Furthermore, patient-controlled analgesia (PCA) options within these systems empower patients to self-titrate doses, enhancing satisfaction and minimizing plasma concentration variability [56]. 

Despite these benefits, continuous neuraxial analgesia is associated with substantial risks, including infection, catheter obstruction, pump malfunction, overdose, and granuloma formation, which may necessitate device explantation. For these reasons, epidural steroid injections are not routinely recommended in the perioperative period [42, 44]. Patients receiving neuraxial therapies require close clinical monitoring to prevent and detect complications. 

## Conclusion

In conclusion, opioid use is intricately linked to both chronic back pain and spine surgery. Although opioids remain a common component of pain management in this population, persistent use is associated with substantial risks, including opioid-induced hyperalgesia, dependence, and fatal overdose.

Spine surgery, while often clinically indicated and effective in improving patient-reported outcomes (PROMs), may paradoxically precipitate new-onset pain due to central sensitization, thereby perpetuating or exacerbating opioid dependence. To mitigate this risk, pain management strategies must increasingly emphasize opioid-sparing alternatives. 

Non-opioid pharmacologic agents and nutraceutical supplements have demonstrated efficacy in improving analgesia and reducing opioid consumption. Regional interventions such as erector spinae plane blocks and neuraxial techniques offer targeted pain control, although their benefits are constrained by short duration of effect (especially with single-shot applications) and procedural risk. Spinal cord stimulation (SCS) may provide additional benefit in select patients, though its effect on long-term opioid reduction remains uncertain. 

 Given these limitations, optimizing postoperative pain control while minimizing opioid reliance remains a complex clinical challenge. A multimodal, interdisciplinary approach—tailored to the individual patient—offers the most promising path forward. As opioids are likely to remain necessary for breakthrough pain in select cases, their use should be carefully restricted, closely monitored, and integrated into structured tapering protocols. Emerging tools such as pharmacogenomic testing may further support personalized prescribing, enhancing safety and sustainability in the management of postoperative spine pain.

## Key references

- Fried TB, Adams A, Ramtin S, Schroeder GD. Evidence-Based Orthopaedic Post-Operative Opioid Prescribing Recommendations Following Spine Surgery. SurgiColl. 2023;1. doi: 10.58616/001c.77650.
  - ◦ Opioid overuse is a major concern in spine surgery, especially for patients with preoperative use or comorbidities. Fried et al provide evidence-based, multimodal regimens to prioritize non-opioid and non-drug strategies, reserving opioids for short-term breakthrough pain to reduce dependence and support stewardship.
- Arciero E, Coury JR, Dionne A, Reyes J, Lombardi JM, Sardar ZM. Optimizing Preoperative Chronic Pain Management in Elective Spine Surgery Patients: A Narrative Review of Outcomes with Opioid and Adjuvant Pain Therapies. JBJS Rev. 2023;11(12). Epub 20231215. doi: e23.00156.
  - ◦ Arciero et al describe how Chronic preoperative opioid use worsens spine surgery outcomes, prolongs recovery, and increases long-term dependence and how preoperative weaning, dose limits, and alternatives such as spinal cord stimulators or intrathecal pumps may help.
- Berardino K, Carroll AH, Kaneb A, Civilette MD, Sherman WF, Kaye AD. An Update on Postoperative Opioid Use and Alternative Pain Control Following Spine Surgery. Orthop Rev (Pavia). 2021;13(2):24978. Epub 20210622. doi: 10.52965/001c.24978. PubMed PMID: 34745473; PubMed Central PMCID: PMC8567777.
  - ◦ The authors provide an overview about the U.S. opioid epidemic that is fueled in part by postoperative prescribing in spine surgery which increases risks of chronic dependence and complications. High-risk factors described include preoperative use, certain patient characteristics, and invasive procedures, while ERAS protocols, multimodal regimens, regional anesthesia, and prescribing limits can reduce opioid use without harming pain control.

## Data Availability

No datasets were generated or analysed during the current study.

## Abbreviations

CPSP: Chronic postsurgical pain
cAMP: Cyclic adenosine monophosphate
ERAS: Enhanced Recovery After Surgery
ICD-11: International Classification of Diseases, 11^th^ revision
NSAID: Nonsteroidal anti-inflammatory drugs
PROMs: Patient-reported outcome measures
SCS: Spinal Cord Stimulation
U.S.: United States
